# Combination TIGIT/PD-1 blockade enhances the efficacy of neoantigen vaccines in a model of pancreatic cancer

**DOI:** 10.3389/fimmu.2022.1039226

**Published:** 2022-12-08

**Authors:** Hui Peng, Lijin Li, Chong Zuo, Michael Y. Chen, Xiuli Zhang, Nancy B. Myers, Graham D. Hogg, David G. DeNardo, S. Peter Goedegebuure, William G. Hawkins, William E. Gillanders

**Affiliations:** ^1^ Department of Surgery, Washington University School of Medicine, St. Louis, MO, United States; ^2^ Department of Medicine, Washington University School of Medicine, St. Louis, MO, United States; ^3^ The Alvin J. Siteman Cancer Center, Washington University School of Medicine and Barnes-Jewish Hospital, St. Louis, MO, United States

**Keywords:** neoantigen, cancer vaccine, pancreatic cancer, combination immunotherapy, TIGIT, checkpoint blockade

## Abstract

**Background:**

Cancer neoantigens are important targets of cancer immunotherapy and neoantigen vaccines are currently in development in pancreatic ductal adenocarcinoma (PDAC) and other cancer types. Immune regulatory mechanisms in pancreatic cancer may limit the efficacy of neoantigen vaccines. Targeting immune checkpoint signaling pathways in PDAC may improve the efficacy of neoantigen vaccines.

**Methods:**

We used KPC4580P, an established model of PDAC, to test whether neoantigen vaccines can generate therapeutic efficacy against PDAC. We focused on two immunogenic neoantigens associated with genetic alterations in the CAR12 and CDK12 genes. We tested a neoantigen vaccine comprised of two 20-mer synthetic long peptides and poly IC, a Toll-like receptor (TLR) agonist. We investigated the ability of neoantigen vaccine alone, or in combination with PD-1 and TIGIT signaling blockade to impact tumor growth. We also assessed the impact of TIGIT signaling on T cell responses in human PDAC.

**Results:**

Neoantigen vaccines induce neoantigen-specific T cell responses in tumor-bearing mice and slow KPC4580P tumor growth. However, KPC4580P tumors express high levels of PD-L1 and the TIGIT ligand, CD155. A subset of neoantigen-specific T cells in KPC4580P tumors are dysfunctional, and express high levels of TIGIT. PD-1 and TIGIT signaling blockade *in vivo* reverses T cell dysfunction and enhances neoantigen vaccine-induced T cell responses and tumor regression. In human translational studies, TIGIT signaling blockade *in vitro* enhances neoantigen-specific T cell function following vaccination.

**Conclusions:**

Taken together, preclinical and human translational studies support testing neoantigen vaccines in combination with therapies targeting the PD-1 and TIGIT signaling pathways in patients with PDAC.

## Introduction

PDAC is currently one of the deadliest cancers and is expected to become the second-leading cause of cancer-related death by 2030 (1, 2). Major factors that are responsible for the poor prognosis of PDAC include the resistance to both chemotherapy and immunotherapy, and the fact that many patients are diagnosed at an advanced stage, or with metastatic disease (2, 3). In terms of cancer immunotherapy, PDAC presents unique therapeutic challenges owing to the dense stroma and immunosuppressive tumor microenvironment (TME) [reviewed in (4)]. Recent studies suggest that the treatment efficacy of immune checkpoint inhibition in PDAC may depend on the presence of high-quality cancer neoantigens and a robust immune infiltrate (5–7).

Neoantigen vaccines have been proposed as a strategy to specifically target cancer neoantigens. Initial clinical trials of neoantigen vaccines for melanoma and glioblastoma have been encouraging (8–10). There are significant conceptual advantages to targeting cancer neoantigens. Neoantigens are expressed exclusively in tumor cells, thereby minimizing the risk of autoimmunity. Neoantigen vaccines can be used to specifically target genetic alterations in cancer driver genes and/or broaden the profile of tumor-specific T cell responses. Nearly all PDAC tumors are predicted to express targetable neoantigens (11). Thus, targeting neoantigens through active vaccination may hold promise as a novel immunotherapy in pancreatic cancer.

High-dimensional profiling of the immune landscape in PDAC demonstrates a deeply immunosuppressive microenvironment. The majority of intratumoral CD8 T cells express a dysfunctional phenotype with elevated surface expression of exhaustion markers. TIGIT is a co-inhibitory receptor expressed on CD4, CD8, and NK cells. PDAC tumor cells express multiple TIGIT ligands such as CD155 and nectins 1 and 4 (12–14) and TIGIT is one of the most common exhaustion markers expressed by intratumoral CD8 T cells (15). Recent studies have demonstrated that TIGIT blockade is capable of restoring T cell function in preclinical models, in particular when combined with PD-1/PD-L1 blockade (12, 16, 17). Restoring T cell function is dependent on the expression of the co-stimulatory receptor, CD226, which competes with TIGIT for binding to CD155 (12, 13). Freed-Pastor et al. further demonstrated that elevated expression of CD155 was found in approximately 80% of human PDAC and immune evasion was maintained through the CD155/TIGIT pathway (18). Therefore, to combat an immunosuppressive pancreatic cancer TME, we hypothesize that a combinatorial strategy comprising (1) neoantigen vaccination to generate neoantigen-specific immune responses, and (2) immune checkpoint blockade of TIGIT/PD-1, is worth pursuing.

To test this hypothesis, we have leveraged the genetically engineered Kras^G12D/+^ Trp53^R172H/+^ p48-Cre (KPC) mouse model. This model recapitulates important aspects of human PDAC, and is commonly used to study human pancreatic cancer (19). Cancer neoantigens have been demonstrated to play an important role in this model. Narayanan et al. studied the KPC4580P cell line derived from a spontaneous tumor in a KPC mouse. They found that irreversible electroporation can serve as an *in situ* vaccine to generate neoantigen-specific T-cell responses (20). We specifically targeted candidate neoantigens identified in KPC4580P using a neoantigen vaccine, and assessed the therapeutic efficacy of combination immunotherapy with TIGIT/PD-1 blockade.

## Materials and methods

### Cell lines

The KPC4580P cell line, derived from a spontaneous tumor in a male LSL-Kras^G12D/+^; LSL-Trp53^R172H/+^; Pdx1^Cre/+^; LSL-Rosa26^Luc/+^ (KPC-Luc) mouse (20), was kindly provided by J.J. Yeh (University of North Carolina at Chapel Hill). KPC4580P cells were cultured in DMEM-F12 medium (Gibco) supplemented with 10% FBS, 2 mM L-glutamine, 1x penicillin/streptomycin (Gibco) at 37°C, 5% CO2. The cell line was tested negative for Mycoplasma.

### Synthetic peptides

Peptides (20- or 25-mer) containing non-synonymous single nucleotide variants were synthesized by GenScript and LifeTein. The peptide sequences (N-C) for the preclinical KPC4580P model are as following: mCAR12(15), ERLVYISFRQGLLT **D** TGLSL; mCDK12(15), SSPFLSKRSLSRSP **I** PSRKS; mCDK12(6), LSRSP **I** PSRKSMKSRSRSPA; mHOOK2(6), LMTKD **A** PDSLSPENYGNFDT; mHPS1(15), RTTGQMVAPSLSPN **K** KMSSE; and the control CMV peptide, GILARNLVPMVATVQGQNLK. Numbers in the parentheses indicate the positions of the mutated amino acids which are also underlined and in bold. In the remainder of this manuscript, mCAR12(15) and mCDK12(15) are simply referred to as mCAR12 and mCDK12, respectively. For the PDAC patient, the three immunogenic peptides are: FOXP3 (p.A439T), AFFRNHPATWKN **T** IRHNLSLHKCFV; FAM129C (p.G520R), RGRVLKKFKSDS **R** LAQRRFIRGWGL; ANK2 (p.R2714H), EEKDSESHLAED **H** HAVSTEAEDRSY. The predicted minimal epitopes with highest affinity for corresponding HLA alleles are underlined.

### Animals and reagents

All animal experiments were approved by the Institutional Animal Care and Use Committee (IACUC) of Washington University in St Louis. Wild-type (WT) C57BL/6 and Rag-1 knockout mice were purchased from The Jackson Laboratories. Rat anti-mouse PD-1 (clone RPM1-14), Rat anti-mouse TIGIT (clone 1G9), MHC class I (clone AF6-88.5.5.3) and class II antibody (clone M5/114), rat anti-mouse CD8 (clone 2.43), and rat anti-mouse CD4 (clone GK1.5) monoclonal antibodies were purchased from Bio X Cell.

### Mouse models

For immunogenicity studies of mutated peptides, age-matched C57BL/6 mice were vaccinated once a week for 2-3 times. The readout was performed five days after the last immunization (see also the Enzyme-linked ImmunoSpot and Flow cytometric analysis method sections). Vaccination was performed by subcutaneous (*s.c.*) injection of 100 μg synthetic peptides and 50 μg Poly IC (InVivoGen) formulated in PBS (100 μl total volume), Poly IC alone as a negative control. For therapeutic tumor experiments, male C57BL/6 mice were inoculated *s.c.* with 5×10^5^ KPC4580P cells into the flank and randomly assigned to treatment groups. Mice were vaccinated (*s.c*.) at the tail base on day 3, 6, 10, 17 and 24. Tumor volume was measured using a caliper and calculated according to the formula (length x width^2^)/2. Mice were then sacrificed at the indicated time points or when the estimated tumor volume reached > 2 cm^3^ (endpoint) or when the tumor was ulcerated.

In some experiments, repeated doses (250 μg per mouse *i.p.*) of anti-CD8 Ab or anti-CD4 Ab were administered to deplete CD8 or CD4 T cells. Successful depletion was confirmed by flow cytometry using PBMC or spleen cells. Depletion was maintained by intraperitoneal administration of the depleting antibody once a week until the end of the study. Peptide vaccination was performed on these mice as described above. In some experiments, 200 μg/dose of anti-PD-1 Ab and 100 μg/dose of anti-TIGIT Ab were administered to the mice (*i.p.*) twice a week.

### Adoptive T cell transfer experiment

Subcutaneous pancreatic tumors were established by implanting 5×10^5^ KPC4580P cells in the right flank of male C57BL/6 mice. Neoantigen vaccinated and Poly IC treated mice were sacrificed at day 35 after tumor inoculation. Splenocytes were isolated and CD3 T cells were purified with the EasySep™ mouse T cell isolation Kit (StemCell). A total of 4×10^6^ CD3 T cells were adoptively transferred into each recipient Rag-1^-/-^ mouse *via i.v.* injection. One day later, 5×10^5^ KPC4580P tumor cells were implanted (*s.c.*) to the right flank of the recipient Rag-1^-/-^ mice. The tumor volume was measured twice a week using a caliper.

### Enzyme-linked ImmunoSpot

After peptide immunization, splenocytes were cultured with or without peptides (4 μg/ml each mCAR12 and mCDK12) overnight at 37°C in pre-coated 96-well plates (Mabtech) and cytokine secretion was detected with an anti-IFN-γ antibody (1 μg/ml, clone R4-6A2, Mabtech). Subtyping of T-cell responses was performed using purified CD3 splenocytes +/**-** MHC class I or class II blocking antibodies (20 µg/ml). All samples were tested in duplicates or triplicates.

### Flow cytometry analysis

Splenocytes were stimulated with peptides (4 μg/ml each mCAR12 and mCDK12) and anti-CD28 (1μg/ml, clone 37.51, BioLegend). Splenocytes treated with anti- CD3 (clone 145-2C11, BioLegend) and anti-CD28 served as positive control. After incubation at 37°C for 2 h, 1 μl/ml of monensin (BioLegend) was added to each sample and incubated at 37°C for an additional 5 h and then held at 4°C overnight. The next day, cells were first stained with live/dead dye followed by staining with appropriate fluorescent antibody cocktails (CD3, CD4, CD8, CD44, CD11a, CD49d, TIGIT, CD226, PD-1) for 30 min on ice. Cells were then fixed and permeabilized using the Foxp3 Cytofix/Cytoperm Buffer Set (eBioscience). Thereafter, cells were stained for IFN-γ, TNF-α and Granzyme B (GzmB) on ice for 30 min. The samples were washed and resuspended in 250 μl of cold PBS containing 2% FBS for analysis using flow cytometry analysis (BD Fortessa X-20 or BD FACScan). Fluorophore conjugated anti-mouse antibodies (clone names in parentheses) used in this study include: from BioLegend, CD11a (M17/4), CD3 (17A2), CD4 (GK1.5), CD4 (RM4-5), CD25 (3C7), CD44 (IM7), CD45 (30-F11), CD49d (R1-2), CD155 (TX56), CD226 (DNAM-1), GzmB (QA16A02), IFN-γ (XMG1.2), TNF-α (MP6-XT22), PD-L1 (10F.9G2), TIGIT (1G9); from BD Biosciences, CD8α (53-6.7); from eBioscience, PD-1 (J43); and from Invitrogen, Foxp3 (FJK-16s). Anti-human antibodies used include: from BioLegend, CD3 (UCHT1), CD8 (RPA-T8), CD11a (HI111), IFN-γ (4S.B3); form eBioscience, CD4 (OKT4); and from BD Biosciences, CD4 (SK3), IFN-γ (B27). Flow cytometry data were analyzed using FlowJo v10 (TreeStar).

To study the tumors, mice were euthanized at day 22 post tumor injection. Portions of harvested tumors were processed using the Mouse Tumor Dissociation Kit (Miltenyi Biotec). The cells were passed through a 70-µm strainer to prepare single-cell suspensions. Cells were stained with live/dead dye followed by staining with appropriate antibody cocktails for 30 min on ice. Intracellular Foxp3 and GzmB staining was performed according to the manufacturer’s protocol (Foxp3 Buffer Set, eBioscience). The samples were washed and resuspended in 250 μl of cold PBS containing 2% FBS for analysis using flow cytometry analysis (BD Fortessa X-20). Data were analyzed using the FlowJo v10 software.

### Patient samples

PBMCs and tumor tissues were collected from pancreatic cancer patients between May 2018 and February 2020 using the Tissue Core funded by the Washington University SPORE in Pancreas Cancer in the Department of Surgery. The patients were diagnosed with resectable PDAC and treated with surgery as the initial treatment modality. Tissue and peripheral blood was collected at the time of surgery. All patients provided informed consent. The study conformed to the principles of the Declaration of Helsinki. The tissue acquisition protocol was approved by the Institutional Review Board at Washington University School of Medicine.

For *in vitro* re-stimulation study using PBMCs from a PDAC patient treated with a polyepitope neoantigen DNA vaccine, 3×10^5^ PBMCs per well were cultured in a 96-well U-bottom plate for three days with 2 µM of each of the three neopeptides (FOXP3, FAM129C, and ANK2, see above) in the presence of recombinant human IL-2 (25 U/ml), anti-CD28 (1 µg/ml, clone CD28.2, BioLegend) and with or without anti-TIGIT antibody (10 µg/ml, clone A15153A, BioLegend). The cells were washed and rested in complete medium supplemented with 2.5 U/ml IL-2 for another three days. The cells were washed again and re-stimulated with the peptide pool (2 µM each) and anti-CD28 (1 µg/ml) for 5 h. Brefeldin-A (GolgiPlug, BD Biosciences) was added for the final 4 h. The cells were harvested and stained for cell surface markers and intracellular cytokines before analyzed by flow cytometry.

### Cytometry by time of flight

Cryopreserved PBMC were thawed in a 37°C water bath and washed in pre-warmed cell culture medium (RPMI-1640, 10% FCS, 1× L-glutamine, and 1× penicillin/streptomycin supplemented with 1:10,000 benzonase (Sigma-Aldrich). Tumor samples were digested at 37°C for 30 min in HBSS (Lonza) supplemented with 2 mg/ml collagenase (Roche), 2.5 U/ml hyaluronidase and DNase I (both from Sigma-Aldrich) to generate a single cell suspension. Reagents including a list of antibodies, detailed procedures of sample preparation, data acquisition and analysis were described previously (21). The data were normalized using the normalization beads and were analyzed using the Cytobank online software.

### Statistical analysis

GraphPad Prism 9 software was used for all statistical analyses. All data are presented as means ± standard error (SEM). Intergroup comparisons were performed using a two-tailed unpaired *t* test, and *P <*0.05 was considered statistically significant. Survival benefits were determined using the log-rank test (Mantel-Cox). To compare three or more groups, we performed one-way ANOVA with Turkeys multiple comparisons test when significant differences were found. *P ≤ 0.05, **P ≤ 0.01, ***P≤ 0.001.

## Results

### Credentialing cancer neoantigens in the KPC4580P pancreatic cancer model

We studied the KPC4580P pancreatic cancer model, which has a mutation burden similar to that of human PDAC (22). Narayanan and colleagues demonstrated that irreversible electroporation (IRE) of KPC4580P tumors induces complete regression in a subset of tumor-bearing animals and the antitumor responses are CD4/CD8 T cell-dependent (20). In the study they performed whole-exome sequencing and RNA sequencing (RNA-seq) to identify KPC4580P neoantigens. ELISpot assay demonstrated that IRE and vaccination with irradiated tumor cells were able to generate T cell reactivity against five peptides (20). To determine the potential of targeting these cancer neoantigens with vaccine therapy, we vaccinated naïve C57BL/6 mice with synthetic long peptides (SLP). The amino acid sequences of the five SLPs (mCAR12, mCDK12(15), mCDK12(6), mHOOK2, and mHSP1) are listed in the Materials and Methods. Vaccination with two of the neoantigen SLPs, namely mCAR12 and mCDK12, was able to generate a response above the background seen in mice vaccinated with adjuvant poly IC alone (**Supplementary Figure S1**). Further analysis of T cells revealed that multifunctional (IFN-γ^+^/TNF-α^+^) neoantigen-specific T cells were detected only in mice vaccinated with the mCAR12/mCDK12 neoantigens and not in control animals (**Supplementary Figures S2A, B**). Both mCAR12 and mCDK12 peptides induced predominantly (but not exclusively) CD4 T cell responses, as the addition of anti-MHC class II antibody completely blocked reactivity to mCDK12 and significantly decreased the number of IFN-γ secreting cells specific to mCAR12 (**Supplementary Figure S2C**). mCAR12 can also stimulate CD8 T cell responses, albeit at a less robust level compared to CD4 T cell responses. We conclude that mCDK12 and mCAR12 are immunogenic neoantigens for the PKC4580P tumor model and focused our study on neoantigen vaccines incorporating these two neoantigens.

### Neoantigen SLP vaccines induce neoantigen-specific CD4 and CD8 T cell responses capable of inhibiting KPC4580P growth

**Figure 1:**
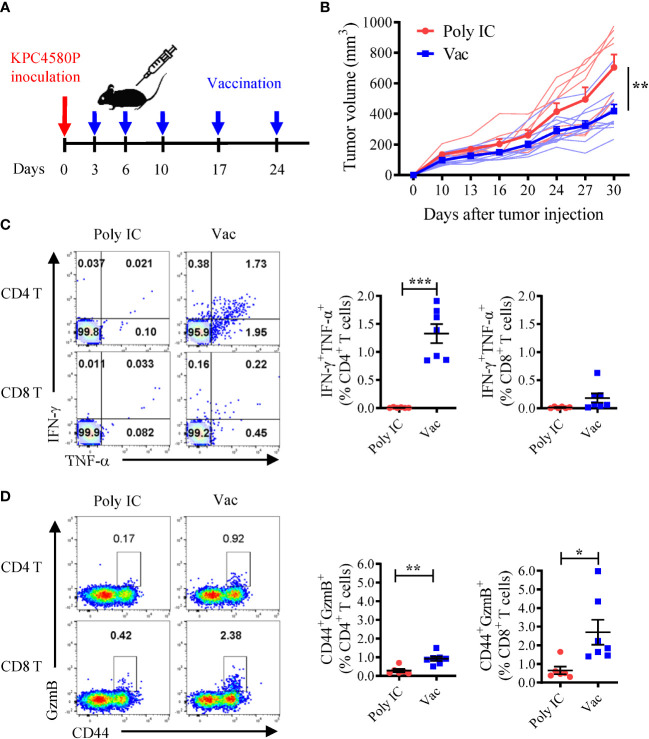
Neoantigen SLP vaccine induces CD4 and CD8 T cell responses and inhibits pancreatic cancer growth. **(A)** Experimental schema. Mice were inoculated with KPC4580P cells followed by vaccination with a neoantigen vaccine incorporating mCAR12/mCDK12 peptides + poly IC (Vac, n = 11) or poly IC alone (Poly IC, n = 8) at the indicated time points. **(B)** Tumor volumes were measured twice a week over time. Individual and mean ± SEM of tumor sizes were plotted. **P < 0.01, *t*-test. **(C)** Neoantigen-specific CD4 and CD8 T cells were analyzed 22 days after tumor inoculation. Spleen cells were stimulated *ex vivo* with a mixture of both mCAR12/mCDK12 peptides, and analyzed by intracellular cytokine staining for IFN-γ and TNF-α. Representative dot plots and summary data are shown. **(D)** Granzyme B expression on CD44^+^ splenic CD4 and CD8 T cells were examined 22 days after tumor inoculation. Data in panel **(C, D)** were derived from one of two similar experiments and were presented as mean ± SEM (n = 6-7). *P < 0.05, **P < 0.01, ***P < 0.001, *t*-test.

To test whether neoantigen-specific T cell responses generated by the mCAR12/mCDK12 neoantigen SLP vaccine could protect mice from KPC4580P tumor challenge, we inoculated mice with tumor cells followed by mCAR12/mCDK12 SLP vaccination (**Figure 1A**). The vaccination was initiated early (three days after tumor inoculation) when tumors were relatively small or not palpable since limited tumor control was achieved if the therapy was delayed until the tumors were large (data not shown). The neoantigen SLP vaccine (Vac) delayed KPC4580P tumor growth (**Figure 1B**). Vaccination was associated with robust mCAR12/mCDK12-specific CD4 T cell responses (**Figure 1C**), and an increase in the number of splenic CD8 and CD4 T cells in vaccinated mice expressing the cytotoxic marker GzmB (**Figure 1D**). Depletion of T cells *in vivo* abrogated the delay in tumor progression observed following vaccination (**Supplementary Figures S3A, B**), suggesting that both CD4 and CD8 T cells contribute to antitumor immunity induced by neoantigen vaccination. To further validate the role of T cells, we isolated splenic T cells from vaccinated tumor-bearing mice and adoptively transferred them into immunocompromised Rag-1^-/-^ mice, followed by tumor challenge (**Supplementary Figure S3C**). The presence of neoantigen-specific T cells was confirmed before transfer by staining for intracellular IFN-γ and GzmB after *ex vivo* stimulation with mCAR12/mCDK12 peptides (**Supplementary Figure S3D**). Tumor growth in Rag-1^-/-^ mice demonstrated a significant reduction in tumor size only when the transferred T cells were obtained from the vaccinated mice (**Supplementary Figure S3E**).

### Neoantigen vaccine increases the number of functional tumor-specific CD4 T cells in the tumor microenvironment

**Figure 2:**
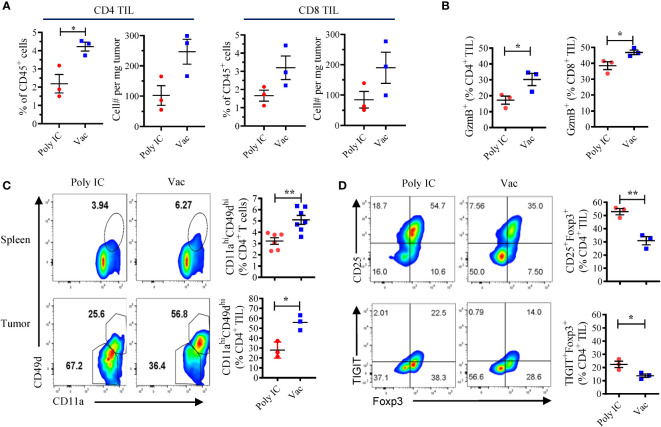
Neoantigen SLP vaccine enhances effector CD4 and CD8 T cells and decreases suppressor CD4 T cells in the tumor microenvironment. **(A)** Flow cytometry analyses of TILs at day 22 after KPC4580P inoculation revealed that treatment with neoantigen SLP vaccine (Vac) is associated with more tumor-infiltrating CD4 and CD8 T cells than control treatment (Poly IC). Percentage of CD4 and CD8 T cells among CD45^+^ cells and the total cell number per mg tumor are shown. **(B)** GzmB expression on CD4 and CD8 TILs at day 22. **(C)** Flow cytometry analysis of CD11a and CD49d among CD4 TIL performed at day 22. **(D)** Foxp3^+^CD25^+^ Treg and TIGIT^+^Foxp3^+^ CD4 T were detected in CD4 TIL at day 22. Significance was determined using *t*-test (n = 3; mean ± SEM; *P < 0.05; **P < 0.01). The experiment was repeated once and similar results were obtained.

Next, we investigated the effect of neoantigen vaccination on T cells in the tumor microenvironment. Tumors in vaccinated mice contained more infiltrating CD4 (4.22 ± 0.42% vs 2.19 ± 0.88%) and CD8 (3.2 ± 1.12% vs 1.66 ± 0.52%) T cells compared to unvaccinated tumors (**Figure 2A**). GzmB expression was also detected in higher percentages of CD4 and CD8 tumor infiltrating lymphocytes (TILs) in vaccinated mice (**Figure 2B**). We chose cell surface expression of integrin CD11a and CD49d as surrogate activation markers for antigen-experienced T cells (23). This approach based on the upregulation of CD49d and CD11a has proven valuable in identifying CD4 and CD8 T cells responding to human vaccines, in particular when there is limited information about the MHC restriction of epitopes/antigens (24). In addition, CD11a also appears to be a useful early activation marker for tumor-specific T cells (25, 26). Both spleen cells and TILs harvested from KPC4580P tumor-bearing mice were stained. We found that in mice vaccinated with neoantigens, compared to mice treated with poly IC alone, a greater percentage of CD4 T cells expressed high levels of CD11a and CD49d, both in spleen and in tumor (**Figure 2C**). Representative gating scheme for CD4 and CD8 TILs is presented in **Supplementary Figure S4A**. Only the CD11a^hi^CD49d^hi^ CD4 T cells, but not the CD11a^lo^CD49d^lo^ subset, produced IFN-γ when stimulated with mCAR12/mCDK12 peptides *in vitro* (**Supplementary Figures S4B, C**), suggesting that CD11a^hi^CD49d^hi^ T cells represent an antigen-experienced subpopulation in the KPC4580P tumor. Taken together, these data demonstrated that neoantigen vaccines result in more tumor-specific T cells with an activated/effector phenotype in the KPC4580P TME.

### Evidence that TIGIT signaling is capable of inducing T cell exhaustion in the KPC4580P tumor model

The inability to completely reject KPC4580P tumors despite the enhanced tumor-specific T cell responses induced by the neoantigen vaccine led us to investigate the potential immune checkpoints. Recently studies have identified a novel CD155/TIGIT axis of inhibition in both murine and human PDAC, and dual TIGIT and PD-1 blockade plus CD40 agonist stimulation was shown to be able to overcome T cell dysfunction in responder mice with established PDAC (18). Therefore we investigated the role of TIGIT in mice challenged with KPC4580P tumors, which express both PD-L1 and the TIGIT ligand CD155, as well as low level MHC class II (**Supplementary Figure S5A**). In KPC4580P tumor bearing mice, TIGIT^+^ T cells were present in spleens and were enriched in the TILs (**Supplementary Figures S5B, C**), indicating a T- cell exhaustion/dysfunctional phenotype. Of note, TIGIT expression was limited to the CD44^+^ memory subset and the expression level increased over time during tumor development (**Supplementary Figure S5B**). Neoantigen vaccination was associated with a decrease in the percentage of regulatory T cells (Treg, CD4^+^CD25^+^FoxP3^+^) and, in particular, TIGIT^+^ Treg in the tumor (**Figure 2D**), likely due to the increased absolute number of tumor-infiltrating CD4 T cells (**Figure 2A**). Although we do not know the mechanisms that lead to the relative reduction in Treg frequency in the tumor, our finding is consistent with other studies that demonstrated a decrease in Treg percentage after neoantigen vaccination (27).

**Figure 3:**
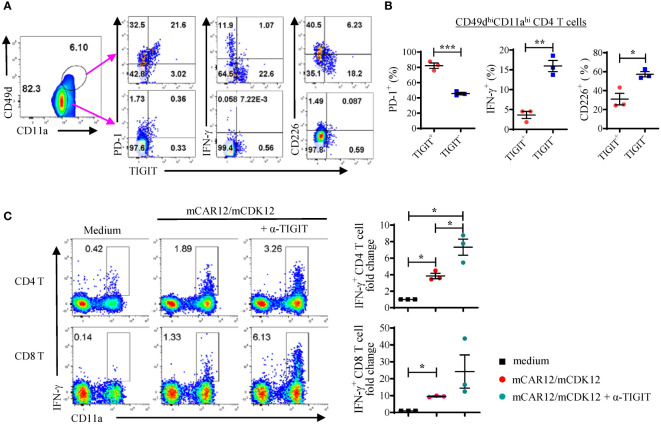
A significant percentage of neoantigen-specific CD4 T cells express high levels of TIGIT. **(A, B)** Splenocytes from vaccinated KPC4580P tumor-bearing mice were stimulated *ex vivo* with a mixture of both mCAR12/mCDK12 peptides and stained with the surface markers CD49d, CD11a, TIGIT, PD-1, CD226, and intracellular cytokine IFN-γ. **(A)** CD4^+^ T cells were gated based on the expression of surrogate markers CD49d and CD11a. Around 24% of the neoantigen-specific CD4 T cells (CD49d^hi^CD11a^hi^) are TIGIT^+^, compared to less than 1% of the CD49d^lo^CD11a^lo^ naïve CD4 T cells. **(B)** Percentages of PD-1, IFN-γ, or CD226 expressing cells were compared between the TIGIT^+^ and TIGIT^-^ populations (CD49d^hi^CD11a^hi^ CD4 cells). **(C)** Splenocytes from vaccinated mice were stimulated with a mixture of both mCAR12/mCDK12 peptides for 3 days in the presence of IL-2 +/- anti-TIGIT Ab and rested for 3 days. On day 6, cells were re-stimulated with a mixture of both mCAR12/mCDK12 peptides for intracellular cytokine staining. Each symbol represents data derived from an individual animal (n = 3; mean ± SEM). *P < 0.05; **P < 0.01; ***P < 0.001, Student *t*-test. Data in **Figure 3** were generated in a single experiment. Similar results were obtained in two additional experiments.

Studies have shown that TIGIT signaling inhibits T cell activation, cytokine production and TCR-mediated T cell proliferation (28). We wanted to investigate whether TIGIT blockade could reverse the TIGIT-mediated exhaustion of neoantigen-specific T cells in response to peptide re-stimulation. In the spleens of KPC4580P tumor bearing mice, the TIGIT^+^ CD4 T cells were mostly found in the antigen-experienced CD11a^hi^CD49d^hi^ cell population (**Figure 3A**) and did not produce IFN-γ after *in vitro* re-stimulation (**Figures 3A, B**). However, when spleen cells from vaccinated tumor bearing mice were stimulated with mCAR12/mCDK12 in the presence of the anti-TIGIT antagonist antibody, more CD4 and CD8 T cells produced IFN-γ as assessed by flow cytometry (**Figure 3C**). These results demonstrate that TIGIT blockade is able to re-activate dysfunctional neoantigen-specific T cells and support the combination of neoantigen vaccine and TIGIT blockade in the treatment of pancreatic cancers.

### Combination TIGIT/PD-1 blockade enhances the ability of neoantigen vaccines to induce antitumor immunity

In mouse tumors, dysfunctional T cells were found to co-express TIGIT and PD-1 (29), and dual blockade of the TIGIT and PD-1 signaling pathways has synergistic effects on intratumoral T cells (30–33). Similarly, we found that in KPC4580P tumor bearing mice, the majority (80%) of the TIGIT^+^ cells also express PD-1 but low levels of CD226 (**Figures 3A, B**). Combining neoantigen vaccine with only anti-PD-1 treatment modestly enhanced KPC4580P tumor protection (**Supplementary Figures S6A, B**). Additional analyses indicated that anti-PD-1 treatment resulted in an increase of TIGIT expression in CD4 and CD8 T cells (**Supplementary Figures S6C, D**). We hypothesized that dual blockade of PD-1 and TIGIT synergizes with neoantigen vaccination in generating optimal anti-tumor immune responses in the KPC4580P pancreatic cancer model.

**Figure 4:**
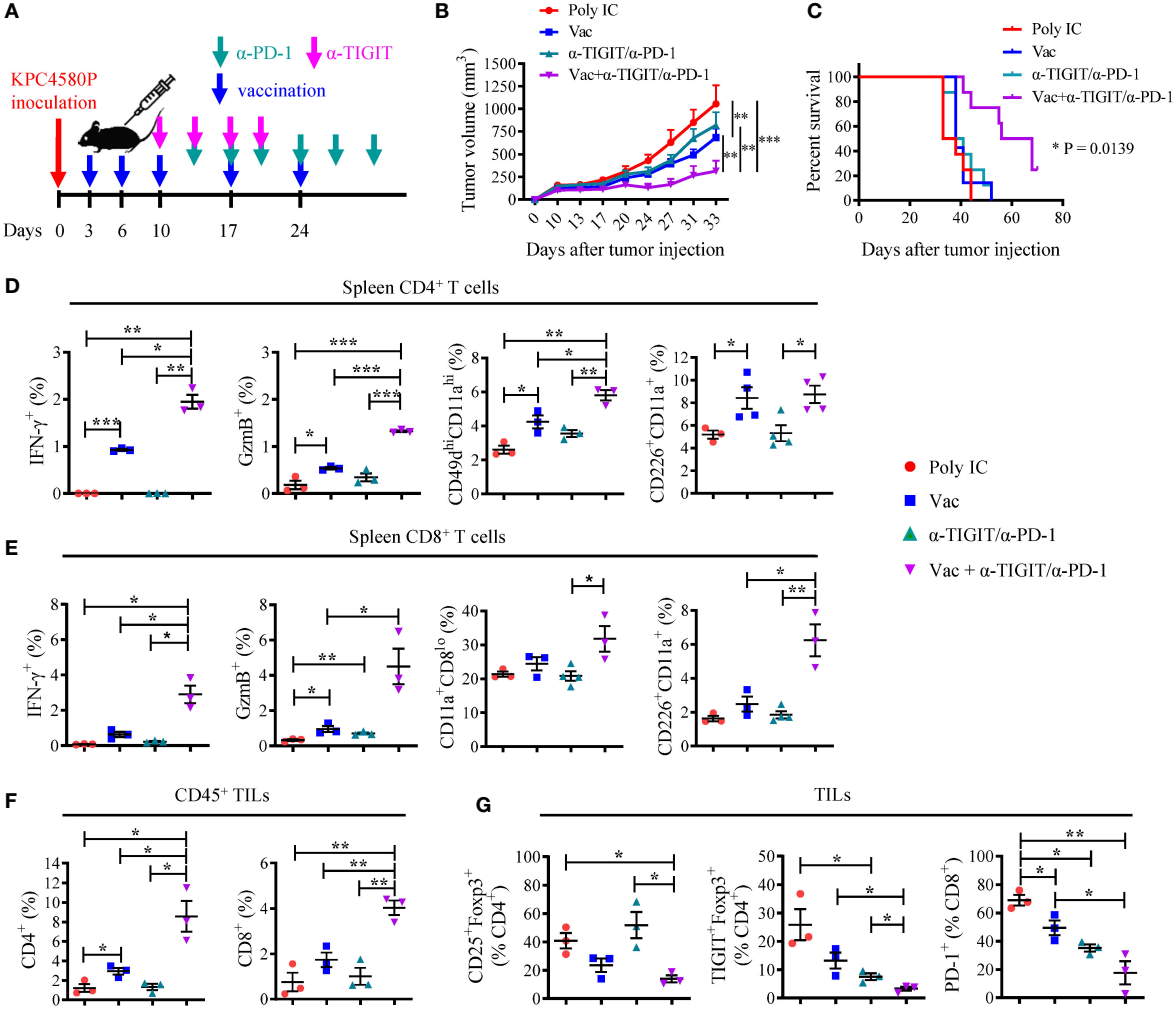
Combination PD-1/TIGIT blockade enhances the response to neoantigen SLP vaccine. **(A)** Treatment timeline for KPC4580P-bearing mice. Three days following KPC4580P implantation, mice were vaccinated with neoantigen SLP followed by anti-TIGIT and anti-PD-1 antibody treatment, as indicated. **(B)** Tumor volumes (mm^3^) were measured twice a week, starting at day 9. Individual tumor growth data can be found in **Supplementary Figure S7A** **(C)** Kaplan-Meier curves showing animal survival rate in each treatment group. *P = 0.0139, Log-rank (Mantel-Cox) test, comparing all 4 groups. **(D, E)** Spleen cells were stimulated *ex vivo* with a mixture of mCAR12/mCDK12 peptides and were analyzed by flow cytometry for the expression of cell surface markers and intracellular molecules. Percentage of populations of cells among gated CD4^+^ **(D)** or CD8^+^ **(E)** T cells were shown. **(F)** Tumor-infiltrating CD4 and CD8 T cells were assessed by flow cytometry. Percentages of CD45^+^ cells that are CD4^+^ and CD8^+^ are shown. Absolute CD4 and CD8 T cell count per mg tumor can be found in **Supplementary Figure S7B**. **(G)** Tumor-infiltrating T cells were harvested and stained for Treg and the surface expression of the exhaustion markers TIGIT and PD-1. Quantitation of CD4^+^ Treg cells and PD-1^+^ CD8 T cells as percentages of total tumor-infiltrating CD4 and CD8 T cells, respectively, are shown. Absolute CD25^+^Foxp3^+^ Treg number per mg tumor mass can be found in **Supplementary Figure S7B**. *P < 0.05, **P < 0.01, ***P < 0.001, unpaired Student’s *t*-test. Representative data from one of three experiments with similar results were shown.

To test the hypothesis, we inoculated C57BL/6 mice with KPC4580P cells at day 0 followed by vaccination starting at day 3. Treatments with anti-TIGIT and anti-PD-1 started at day 10 and day 13, respectively (see **Figure 4A** for detailed treatment schema). The tumor size was measured twice per week. Although TIGIT and PD-1 dual blockade alone did not seem to impact tumor growth, combining neoantigen vaccine substantially suppressed tumor growth (**Figure 4B** and **Supplementary Figure S7A**) and led to longer survival of tumor-bearing animals (**Figure 4C**). In addition, combination therapy also had a significant impact on the number and phenotype of neoantigen-specific T cells in the spleen and tumor microenvironment. Compared to vaccine alone, combination therapy resulted in a higher percentage of splenic CD4 and CD8 T cells that produce IFN-γ and GzmB in response to neoantigen re-stimulation (**Figures 4D, E**). The frequency of effector splenic CD4 T cells (CD11a^hi^CD49d^hi^, CD226^+^) also increased in mice receiving combination therapy (**Figure 4D**). It is worth noting that although neoantigen vaccination alone did not induce a robust CD8 T cell response, dual TIGIT/PD-1 blockade was able to significantly enhance the percentage of CD226^+^ CD8 T cells and neoantigen-specific CD8 T cell response (**Figure 4E**). There were also more CD4 and CD8 TILs in tumors treated with combination therapy as compared to those treated with either neoantigen vaccine, or anti-TIGIT/anti-PD-1 antibodies alone (**Figure 4F** and **Supplementary Figure S7B**). Even though all CD4 T cell subsets increase following vaccination including Tregs when normalized using cell count per mg tumor (data not shown), some T cell subsets expand much more than others. We found that the CD4eff/Treg and CD8/Treg ratios increased following neoantigen vaccination (**Supplementary Figure S7C**). As a result, there were lower percentages of Tregs (in particular, TIGIT^+^ Tregs) and PD-1^+^ CD8 T cells in the tumors treated with combination therapy (**Figure 4G**). These data demonstrate that dual PD-1/TIGIT blockade enhances immune responses induced by neoantigen vaccine, which results in superior antitumor immunity.

### TIGIT expression and evidence of TIGIT signaling in patients with pancreatic cancer

**Figure 5:**
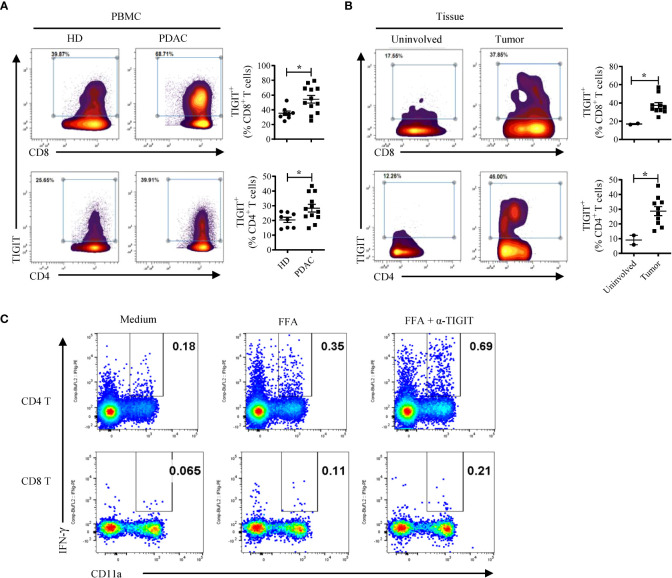
TIGIT restrains T cell responses in human PDAC. **(A)** CyTOF analysis of TIGIT expression in human CD4 and CD8 T cells in the PBMCs from PDAC patients (n = 12) and healthy donors (HD, n = 8). **(B)** CyTOF analysis of TIGIT expression in human CD4 and CD8 T cells isolated from tumors (n = 10) and uninvolved tissues (n = 2) of PDAC patients. Each dot represents data from an individual human subject. Data were presented as Mean ± SEM. *P < 0.05, Mann-Whitney tests. **(C)** PBMCs from a PDAC patient vaccinated with neoantigen DNA vaccine were cultured with a mix of 3 neopeptides (FFA) plus IL-2 for 3 days with or without the anti-TIGIT antibody. The cells were rested for 3 days followed by FFA re-stimulation and analyzed by intracellular cytokine staining and flow cytometry. A similar two-fold increase in IFN-γ producing CD4 and CD8 T cells was obtained when anti-TIGIT antibody was added to the culture of PBMCs from the same patient that were re-stimulated with the viral CEF peptide pool (data not shown).

To extend these findings, we investigated whether TIGIT signaling is an important immune regulatory pathway in human pancreatic cancer. To do this, we first examined the expression of TIGIT in peripheral blood and tumor specimens derived from PDAC patients. CyTOF analyses indicated that TIGIT expression is increased on peripheral CD4 and CD8 T cells in human PDAC (**Figure 5A**). We also compared TIGIT expression in T cells isolated from human PDAC (n = 10) and a limited number (n = 2) of adjacent uninvolved tissue and noted a significantly higher TIGIT expression in tumor tissues than in uninvolved adjacent tissues (**Figure 5B**). These findings are in agreement with a recent report that human pancreatic cancer has an increased TIGIT protein expression on T and NK cells (15).

To test whether TIGIT signaling blockade can reinvigorate T cell responses in patients with pancreatic cancer, we added anti-TIGIT Ab to *in vitro* T cell cultures using PBMCs from a pancreatic cancer patient treated with a polyepitope neoantigen DNA vaccine on an expanded access protocol. The DNA vaccine was constructed as described previously (34) and was manufactured in the GMP facility at WUSM. The neoantigen DNA vaccine was administered monthly using an integrated electroporation device. A total of 14 neoantigens were targeted, and neoantigen-specific T cell responses were detected against three neoantigens (FOXP3 (p.A349T), FAM129C (p.G520R), and ANK2 (p.R2714H). To determine whether the TIGIT blockade is capable of reversing any potential neoantigen-specific T cell exhaustion, we stimulated post-vaccine PBMCs with a mix of the three neoantigen peptides plus IL-2 for 3 days with or without the anti-TIGIT antibody. The cells were rested for 3 days followed by peptide re-stimulation and analyzed by intracellular cytokine staining and flow cytometry (**Figure 5C**). The results show a roughly 2-fold increase in IFN-γ producing CD4 and CD8 T cells when anti-TIGIT antibody was included in the initial 3-day culture. Although we have only tested a single patient sample, these data suggest that blocking TIGIT signaling has the potential to reverse T cell dysfunction and provide support for further investigation of the combination of neoantigen vaccine and anti-TIGIT immunotherapy in human PDAC patients.

## Discussion

Neoantigens are known to be important targets of anti-tumor T cell responses. Here we generated a neoantigen vaccine comprising two 20-mer SLPs identified in the KPC4580P pancreatic cancer model. The neoantigen SLP vaccine was able to induce neoantigen-specific T cells in mice and reduce tumor growth. In combination with PD-1/TIGIT blockade, neoantigen vaccination resulted in enhanced tumor regression. This study provides support for combination therapy using neoantigen vaccines plus immune checkpoint inhibition targeting PD-1/TIGIT in pancreatic cancer patients.

Recent studies in three preclinical tumor models indicated that CD4 T cells play an important role in tumor control (35). Our findings are in line with this published report; the two neoantigens in our study elicited predominantly CD4 T cells. Our model provides potential insights into the function the neoantigen-specific CD4 T cells. We used the surrogate activation markers CD11a and CD49d to assess the T cell responses in tumors. Expression of CD11a was initially used to track antigen-primed effector and memory T cells induced by viral vaccination (36), but more recently, Liu et al. demonstrated that high expression of CD11a can also be used as a marker to identify and track endogenous tumor reactive CD8 T cells (25). We found that neoantigen vaccinated tumor-bearing mice display more CD11a^hi^CD49d^hi^ CD4 T cells and lower percentage of Tregs in the TME compared to vehicle-treated tumor-bearing mice. The CD11a^hi^CD49d^hi^ CD4 T cells in vaccinated mice comprised the majority of IFN-γ- and GzmB-producing cells (**Figure 4**). Of note, we have not examined the CD11a^hi^CD49d^lo^ T cell population for their functionality and neoantigen-specificity. It is possible that the CD8 T cell response induced following vaccination may not be entirely mCAR12 and mCDK12 specific. A recent study using a *Plasmodium* infection model indicated that activated CD4 T cells develop into both CD11a^hi^CD49d^hi^ type 1 helper T (Th1) cells and CD11a^hi^CD49d^lo^ follicular helper T (Tfh)-like cells (37). The exact mechanism through which neoantigen-specific CD4 T cells mediate tumor regression is unknown at this point. The elevated level of GzmB in CD4 TIL compared to CD4 splenocytes indicates cytolytic function. It is possible that CD4 TIL can directly mediate tumor cell killing. We found that at baseline a small percentage (14.4%) of KPC4580P tumor cells express low levels of MHC class II. Upon IFN-γ treatment, the percentage of KPC4580P cells that express MHC II increases to 25.2-28.2% (**Supplementary Figure S5A**). Further studies, perhaps through a modification of our adoptive T cell transfer experiment in tumor-bearing Rag^-/-^ mice (see **Supplementary Figure S3**) using CD4 T cells from perforin/GzmB knockout mice could provide further details on the exact mechanism. Given the dependence on CD8 T cells for tumor control (**Supplementary Figure S3**), our data overall suggest that neoantigen vaccination induces specific CD4 T cells, and expands, and broadens the tumor-directed T cell response including neoantigen-specific CD8 T cells. However, the CD8 response induced by neoantigen vaccination is likely restrained by the upregulation of immune checkpoint molecules such as PD-1 and TIGIT. We demonstrated that dual blockade of TIGIT and PD-1 can enhance the CD8 T cell response to neoantigen vaccines. Of note, neoantigen-specific CD4 T cells have been identified in several neoantigen vaccine studies (9, 35) including our own neoantigen DNA vaccine trial in TNBC (38). Neoantigen-reactive CD4 T cells have also been shown to mediate clinical regression in a patient with cholangiocarcinoma when neoantigen-reactive CD4 T cells were adoptively transferred (39), further confirming the important contribution of neoantigen-specific CD4 T cells towards antitumor immunity.

As has been described in multiple reports, intratumoral CD8 T cells in PDAC display an exhausted phenotype, typified by the expression of TIGIT and frequently of PD-1. Our data extend these findings, demonstrating that TIGIT^+^ CD4 T cells express higher levels of PD-1, less CD226, and produce less IFN-γ than TIGIT^-^ CD4 T cells (**Figures 3A, B**), suggesting a dysfunctional phenotype of the TIGIT^+^ CD4 T cells. While neoantigen vaccination or TIGIT blockade partially restored immune function, our study also suggested that neoantigen vaccination (and possibly anti-TIGIT blockade) could also result in increased expression of the PD-1/PD-L1 pathway (**Figure 3C** and **Supplementary Figure S5**) (7), possibly through activated effector T cells producing IFN-γ. Indeed, our data showed that exposure of KPC4580P tumor cells to IFN-γ greatly increased PD-L1 expression, with the potential to bind to PD-1 on neoantigen activated T cells leading to T cell dysfunction (6). Our data also show that combination therapy of neoantigen vaccine plus anti-PD-1 modestly enhanced tumor protection which may be related to the observation that PD-1 treatment increased TIGIT expression in T cells (**Supplementary Figure S6C**). This finding is consistent with a study on hepatocellular cancer showing anti-PD-1 therapy greatly upregulated TIGIT expression in activated T cells and the CD155/TIGIT axis contributed to anti-PD-1 treatment resistance (30). Additionally, recent studies demonstrated that the CD155/TIGIT axis is a key driver of immune evasion in pancreas cancer (18), and that both PD-1 and TIGIT signaling impairs CD226 co-stimulation (17) which is required for restoring antitumor immunity. Based on this, and our observation that CD226 is readily expressed on neoantigen-specific T cells after vaccination (**Figures 4D, E**), we treated tumor-bearing mice with combination PD-1/TIGIT blockade and neoantigen vaccine. This combination not only improved vaccine-induced T cell responses, but also enhanced T cell infiltration in the tumor.

Of note, combination PD-1/TIGIT blockade has entered clinical testing. In patients with NSCLC, combination therapy showed meaningful improvement in response rate and progression-free survival (40–42). However, combination PD-1/TIGIT blockade using the same antibodies plus chemotherapy did not meet the primary endpoints of progression-free survival and overall survival in patients with extensive-stage SCLC (43). It is likely that the benefit of combination PD-1/TIGIT therapy will be dependent on the cancer type and clinical context. In this context, TIGIT blockade appears highly relevant in patients with PDAC. Steele et al. showed that TIGIT expression is increased on T and NK cells in pancreatic cancer and its expression in the tumors correlates with its expression in matched blood (15). Our CyTOF data showed TIGIT expression is increased in both CD4 and CD8 T cells compared to healthy donors and higher TIGIT expression was found on immune cells from PDAC tumors compared to uninvolved tissue. The present study suggests that targeting the PD-1 and TIGIT signaling pathways enhances the response to neoantigen vaccines in pancreatic cancer, highlighting the potential synergy of these therapies in pancreatic cancer.

## Data availability statement

The original contributions presented in the study are included in the article/**Supplementary Materials**. Further inquiries can be directed to the corresponding author/s.

## Ethics statement

The studies involving human participants were reviewed and approved by the Institutional Review Board at Washington University School of Medicine. The patients/participants provided their written informed consent to participate in this study. The animal study was reviewed and approved by the Institutional Animal Care and Use Committee (IACUC) of Washington University in St Louis.

## Author contributions

HP, SG, WH, and WG contributed to conception and design of the study. HP, LL, CZ, MC, XZ, NM, and GH performed experiments. HP, LL, SG, WH, and WG analyzed data. DD, WH, and WG supervised the study. HP and LL wrote the manuscript. LL, SG, and WG edited the manuscript. All authors contributed to the article and approved the submitted version.
