# Supplementary material for: Combination TIGIT/PD-1 blockade enhances the efficacy of neoantigen vaccines in a model of pancreatic cancer
Source: Front Immunol. 2022 Dec 8;13:1039226. doi: 10.3389/fimmu.2022.1039226 (PMC9772034; doi:10.3389/fimmu.2022.1039226)
Supplement: Supplementary file 1 [file DataSheet_1.pdf]

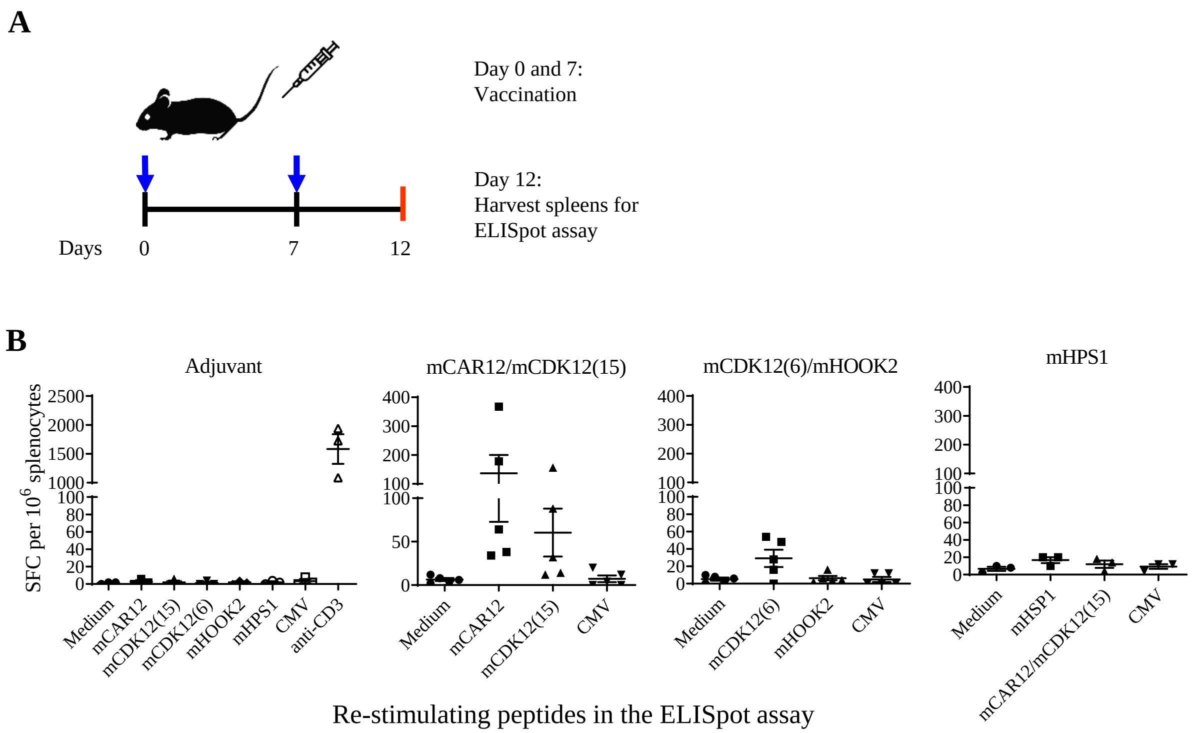

Figure S1. KPC4580P neoantigens mCAR12 and mCDK12 are immunogenic. (A) Schematic experimental design. C57BL/6 mice (n= 5 each group) were vaccinated twice on day 0 and day 7. Five days later (day 12), spleens were harvested and single-cell suspensions were prepared for *ex vivo* IFNγ ELISpot. **(B)** ELISpot assay results indicated that peptide vaccination with mCAR12 and mCDK12(15) was able to induce neoantigen-specific immune response *in vivo*. Adjuvant (Poly IC) alone, or peptide pools (100 μg each) used as vaccines were indicated above each plot. Each symbol represents data from an individual animal.

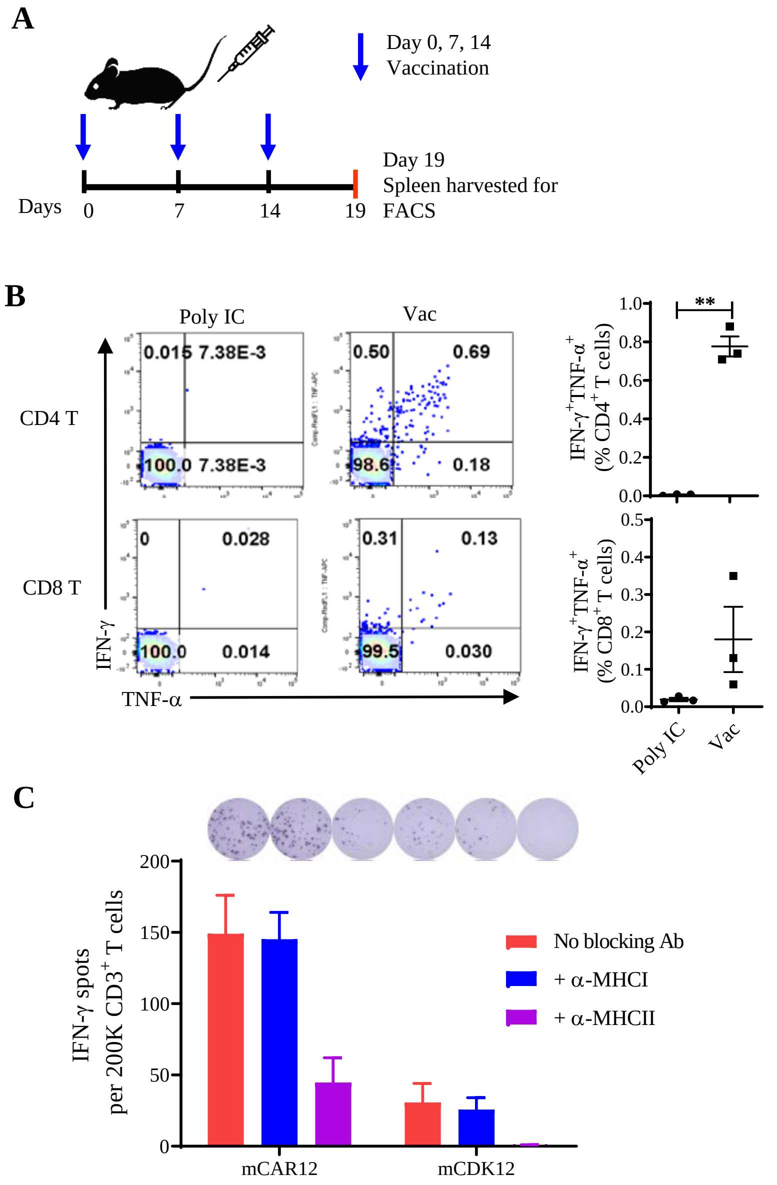

Figure S2. mCAR12/mCDK12 neoantigen vaccine elicits T cell responses. (A) Experimental schedule. C57BL/6 mice were vaccinated three times with a mix of 100 µg each mCAR12 and mCDK12, or Poly IC alone (n= 3 each group). Five days after the final vaccination, spleens cells were prepared and stimulated *ex vivo* with mCAR12 and mCDK12 peptides. **(B)** Peptide-specific IFN-γ and TNF-α production was measured by intracellular cytokine staining and flow cytometry. **(C)** $CD3^+$ T splenocytes from vaccinated mice were used in an IFN-γ ELISpot assay with or without the addition of MHC class I or MHC class II -blocking antibody (20 µg/ml). Numbers of spots in response to 20-mer mCAR12 or mCDK12 peptides were shown.

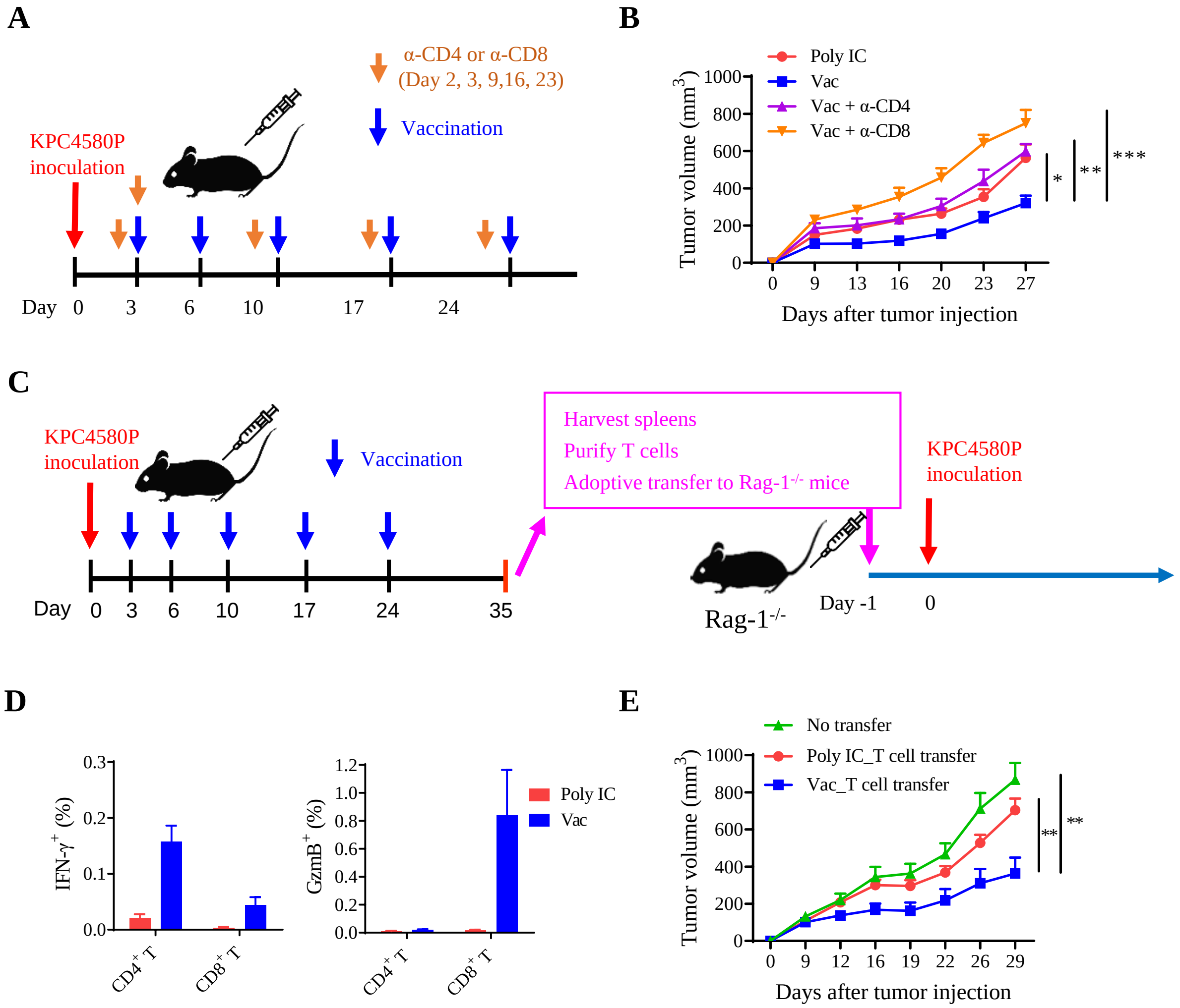

Figure S3. Tumor regression induced by neoantigen vaccine requires both CD4 and CD8 T cells. (A) Experimental schema of T cell deletion. Anti-CD4 or anti-CD8 depleting antibodies were administered (*i.p.*) before vaccination and throughout the study (n= 7 or 8 each group). **(B)** Tumor volume measured over time after KPC4580P cells inoculation. **(C)** Experimental schema of T cell adoptive transfer study. KPC4580P tumor-bearing mice were vaccinated with neoantigen mCDK12/mCAR12 or poly IC alone as indicated. At day 35, $CD3^+$ T cells were isolated from spleens and were adoptively transfer (4×10^6 T cells per recipient mouse) into immunocompromised Rag-$1^{-/-}$ mice followed by tumor challenge one day later (n= 6 or 7 each group). **(D)** IFNγ- and GzmB -producing T cells were detected in donor spleens by intracellular cytokine staining after in vitro stimulation with mCAR12/mCDK12 peptides. **(E)** KPC4580P tumor growth in Rag-$1^{-/-}$ mice received T cell adoptive transfer.

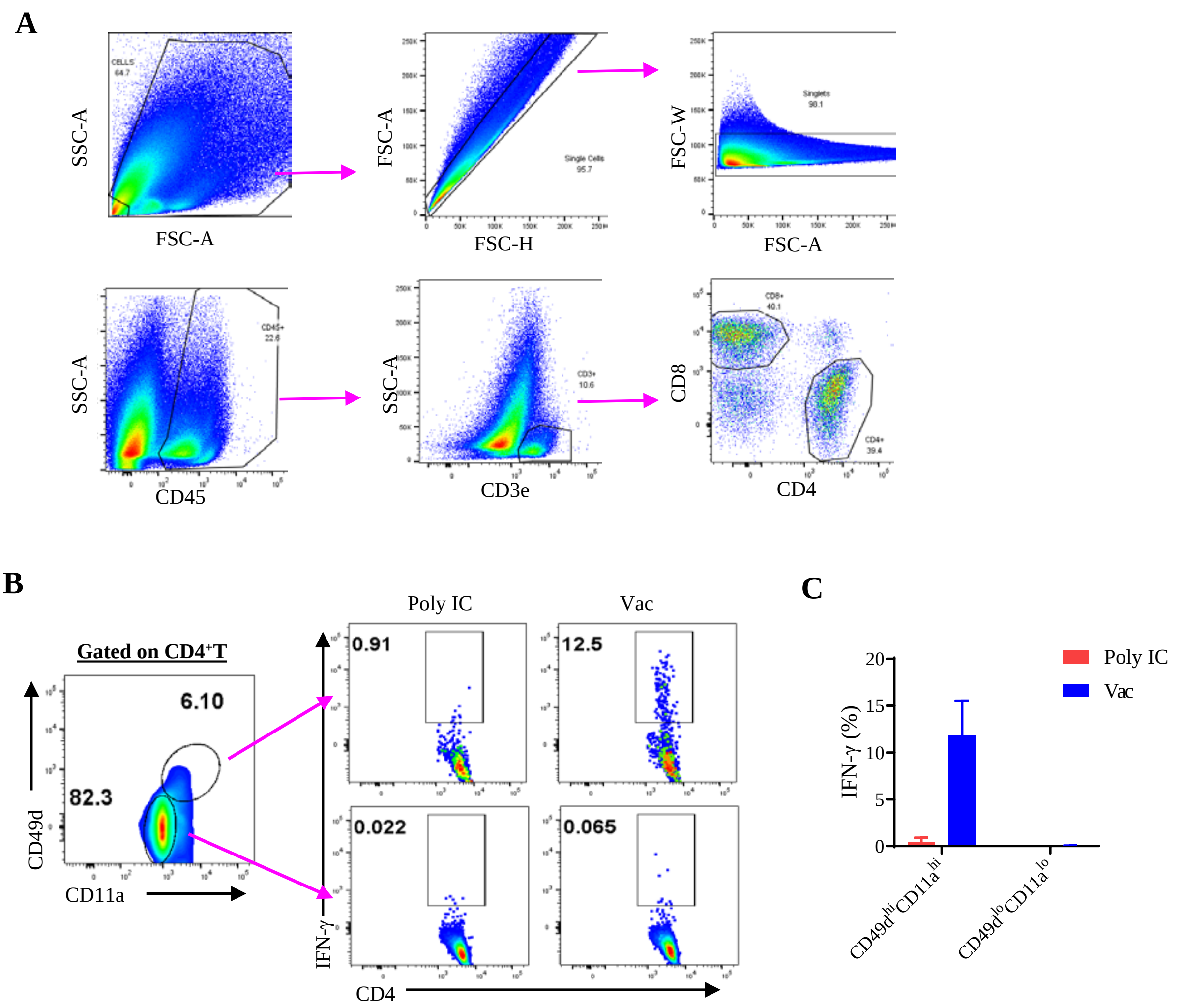

Figure S4. CD49d^{hi}CD11a^{hi} surrogate markers identify a CD4 effector T cell subpopulation in KPC4580P tumor bearing mice. (A) Representative gating strategy used in flow cytometry data analysis to identify the CD4 and CD8 TILs. **(B)** Representative plots showing CD11a, CD49d and IFN-γ staining on CD4 T cells after *ex vivo* re-stimulation with mCAR12/mCDK12 peptides. Spleen cells were harvested at day 22 from KPC4580P tumor-bearing mice vaccinated with neoantigens mCAR12/mCDK12 or Poly IC alone. **(C)** Collective data showing frequencies of IFN-γ-producing cells by CD49d^{hi}CD11a^{hi} and CD49d^{lo}CD11a^{lo} CD4 T cells after stimulation with mCAR12/mCDK12.

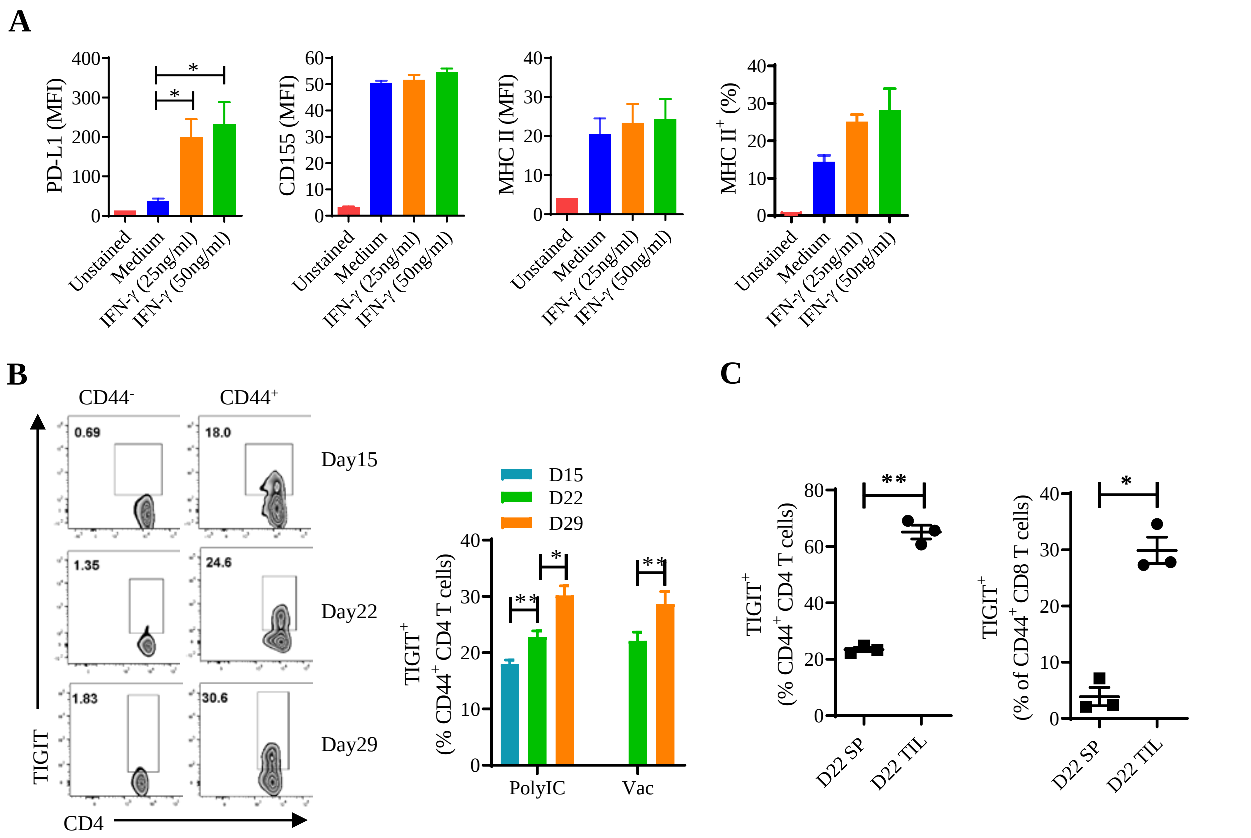

Figure S5. TIGIT expression in T cells increases during tumor development. (A) The mean fluorescence indexes (MFI) indicate the expression levels of PD-L1, CD155 and MHC class II on cultured KPC4580P cells with or without IFN-γ treatment for 24 h. Percentages of MHC II+ cells were also shown. **(B)** Flow cytometric analysis of TIGIT expression in $CD44^-$ and $CD44^+$ CD4 T cells in spleens from KPC4580P tumor bearing mice at day 15, 22, 29 after tumor injection. Bar graph summarizes data from 3-4 animals generated at each time point. **(C)** Percentage of $TIGIT^+$ cells among $CD44^+$ CD4 and CD8 T cells from the spleens (SP) and TIL of KPC4580P tumor bearing mice at day 22. $^*P < 0.05$ and $^{**}P < 0.01$, student *t*-test.

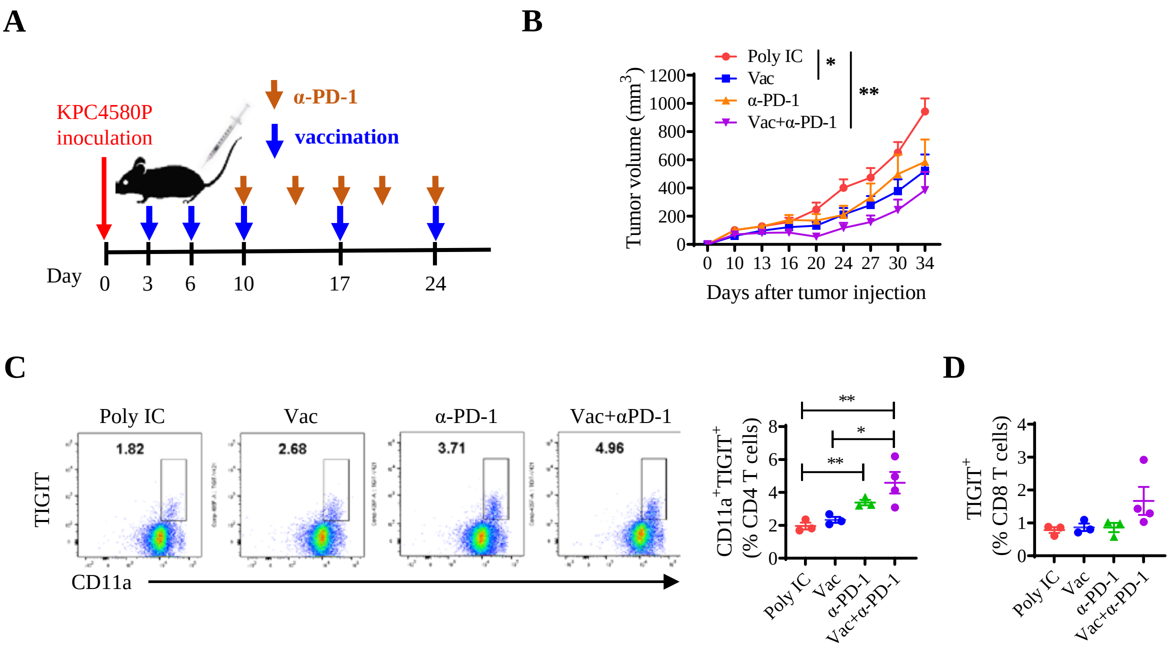

Figure S6. PD-1/PD-L1 blockade upregulates TIGIT expression on T cells. (A) Treatment timeline for KPC4580P-bearing mice. Three days following KPC4580P implantation, mice received Vac (100 µg each mCDK12 and mCAR12). Anti-PD-1 antibody (200 µg) was administration twice a week as shown starting at day10. **(B)** Tumor volumes was measured every 3 to 4 days. Student's *t*-test was performed using measurements collected at day 34. $*P < 0.05$, $**P < 0.01$. **(C-D)** Flow cytometry analysis of TIGIT expression on CD4 T cells (**C**) and CD8 T cells (**D**) from the spleens of KPC4580P tumor bearing mice at day 22. Unpaired *t*-test, $*P < 0.05$, $**P < 0.01$.

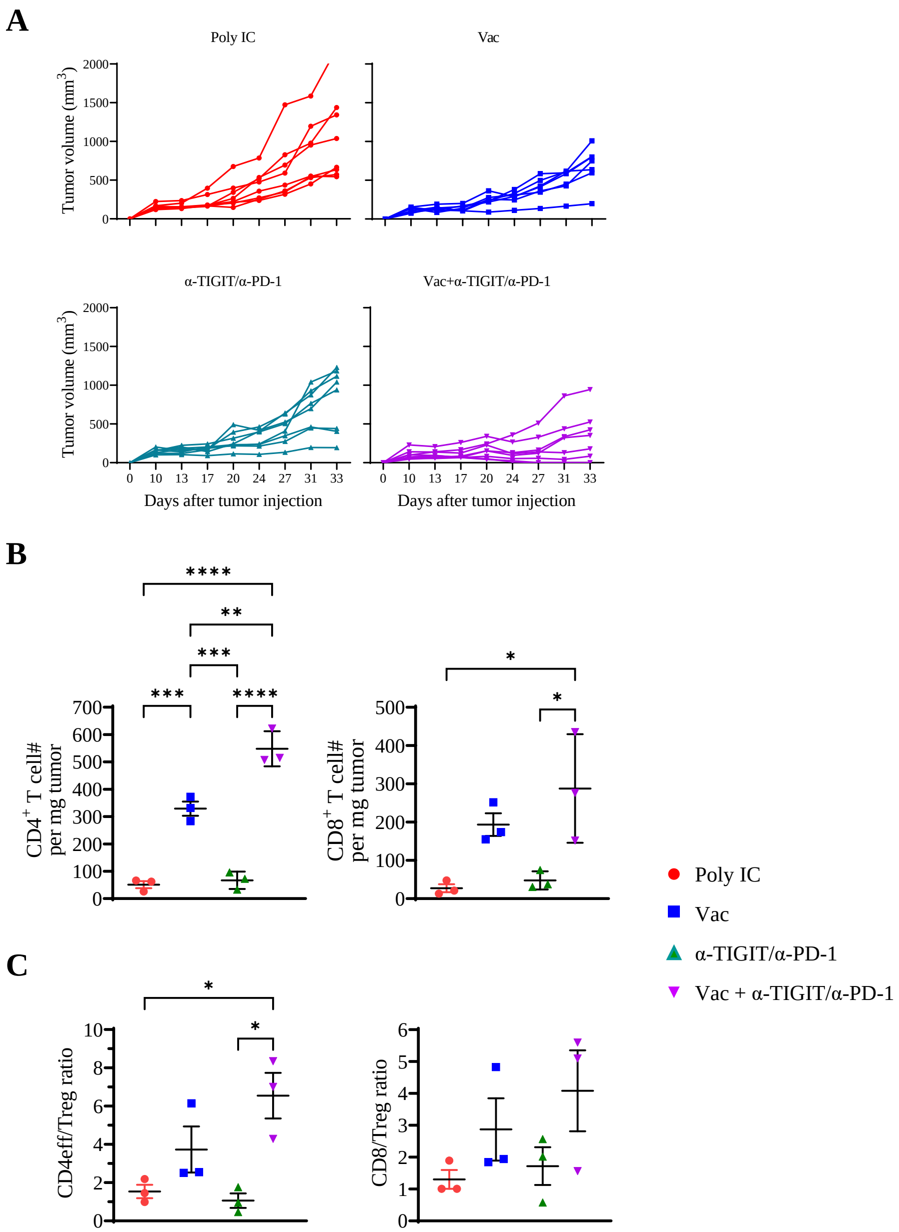

Figure S7. PD-1/TIGIT dual blockade enhances the response to neoantigen SLP vaccine. **(A)** Individual KPC4580P tumor growth data corresponding to **Figure 4B**. Mice were inoculated with KPC4580P tumor cells. Three days later, tumor-bearing mice were vaccinated with neoantigen SLP followed by anti-TIGIT and anti-PD-1 antibody treatment, as indicated. Individual tumor sizes (mm^3) were measured twice a week. **(B)** Number of CD4 and CD8 T cells per mg of KPC4580P tumors at day 22 after indicated treatments. (**C**) CD4eff/Treg and CD8/Treg ratios of TILs in the KPC4580P tumors after indicated treatments. Each symbol indicates data from an individual animal. Ordinary one-way ANOVA multiple comparisons were performed for statistical significance, ** $P < 0.01$, *** $P < 0.001$, **** $P < 0.0001$.
